# Effects of Field Position on Fluid Balance and Electrolyte Losses in Collegiate Women’s Soccer Players

**DOI:** 10.3390/medicina56100502

**Published:** 2020-09-24

**Authors:** Haoyan Wang, Kate S. Early, Bailey M. Theall, Adam C. Lowe, Nathan P. Lemoine, Jack Marucci, Shelly Mullenix, Neil M. Johannsen

**Affiliations:** 1 College of Physical Education and Health Sciences, Zhejiang Normal University, Jinhua 321000, China; hwang56@lsu.edu; 2 School of Kinesiology, Louisiana State University, Baton Rouge, LA 70803, USA; early_kate@columbusstate.edu (K.S.E.); btheal1@lsu.edu (B.M.T.); alowe19@lsu.edu (A.C.L.); nlemoi3@lsu.edu (N.P.L.J.); 3 Department of Kinesiology & Health Sciences, Columbus State University, Columbus, GA 31907, USA; 4 Department of Athletics, Louisiana State University, Baton Rouge, LA 70803, USA; jmarucc@lsu.edu (J.M.); smulle1@lsu.edu (S.M.)

**Keywords:** women athletes, soccer, sweat electrolytes, fluid balance, sweat rate

## Abstract

*Background and objectives*: Research investigating hydration strategies specialized for women’s soccer players is limited, despite the growth in the sport. The purpose of this study was to determine the effects of fluid balance and electrolyte losses in collegiate women’s soccer players. *Materials and Methods*: Eighteen NCAA Division I women’s soccer players were recruited (age: 19.2 ± 1.0 yr; weight: 68.5 ± 9.0 kg, and height: 168.4 ± 6.7 cm; mean ± SD), including: 3 forwards (FW), 7 mid-fielders (MD), 5 defenders (DF), and 3 goalkeepers (GK). Players practiced outdoor during spring off-season training camp for a total 14 practices (WBGT: 18.3 ± 3.1 °C). The main outcome measures included body mass change (BMC), sweat rate, urine and sweat electrolyte concentrations, and fluid intake. *Results*: Results were analyzed for comparison between low (LOW; 16.2 ± 2.6° C, *n* = 7) and moderate risk environments for hyperthermia (MOD; 20.5 ± 1.5 °C, *n* = 7) as well as by field position. The majority (54%) of players were in a hypohydrated state prior to practice. Overall, 26.7% of players had a %BMC greater than 0%, 71.4% of players had a %BMC less than −2%, and 1.9% of players had a %BMC greater than −2% (all MD position). Mean %BMC and sweat rate in all environmental conditions were −0.4 ± 0.4 kg (−0.5 ± 0.6% body mass) and 1.03 ± 0.21 mg·cm^−2^·min^−1^, respectively. In the MOD environment, players exhibited a greater sweat rate (1.07 ± 0.22 mg·cm^−2^·min^−1^) compared to LOW (0.99 ± 0.22 mg·cm^−2^·min^−1^; *p* = 0.02). By position, DF had a greater total fluid intake and a lower %BMC compared to FW, MD, and GK (all *p* < 0.001). FW had a greater sweat sodium (Na+) (51.4 ± 9.8 mmol·L^−1^), whereas GK had the lowest sweat sodium (Na+) (30.9 ± 3.9 mmol·L^−1^). *Conclusions*: Hydration strategies should target pre-practice to ensure players are adequately hydrated. Environments deemed to be of moderate risk of hyperthermia significantly elevated the sweat rate but did not influence fluid intake and hydration status compared to low-risk environments. Given the differences in fluid balance and sweat responses, recommendations should be issued relative to soccer position.

## 1. Introduction

Competitive soccer players move continuously during match play, covering large distances (8–12 km) using both aerobic and anaerobic energy systems [1,2]. Due to the nature of the sport, soccer players have limited access to fluids and time to rehydrate as there are no timeouts and minimal player substitutions [2]. The requirements of high intensity exercise combined with limited fluid availability puts players at a high risk of hypohydration, which can be further exacerbated by warm/hot and humid environmental conditions [3,4]. Fédération Internationale de Football Association (FIFA) declares hydration breaks at the 30th and 75th min of each 90 min match when environmental stress, assessed via wet-bulb globe temperature (WBGT), is greater than 31 °C. In addition, according to the new rules proposed by the National Collegiate Athletic Association (NCAA) in 2019, players are required to take hydration breaks between 25–30 min and 70–75 min when WBGT reaches 30 °C. Without these hydration breaks on hot/humid days, the loss of body water and electrolytes may significantly alter cardiovascular function and thermoregulation, and impair athletic performance [5,6]. Determining an individual’s body mass change (BMC) is considered the simplest method to assess fluid balance and hydration status in sport [7]. In fact, past research has shown that ≥−2% BMC is frequently observed in competitive soccer and is associated with player’s risk of impaired physical and cognitive performance [8], heat-related illness, and injury [9,10].

Current hydration guidelines exist to protect players from illness and injury as a result of hypohydration. Hydration requirements vary across each sport, as well as the position within each sport due to differences in exercise mode and intensity, and environmental conditions [11]. Research has emphasized that hydration guidelines should be both sport- and sex-specific due to known differences in drinking behavior and fluid balance [6,11]. As women’s soccer continues to grow across all competitive levels, additional research aimed at the development of specific hydration guidelines for women is warranted, especially considering hypohydration tends to be more prevalent in women’s soccer [12,13,14]. 

A recent publication by Belval et al. (2019) suggested that personalized hydration strategies are necessary to promote adequate hydration and optimize performance based on the exercise intensity, fluid availability, and environmental condition of each sport [4]. In competitive soccer, players exhibit different exercise characteristics (sprinting, running, walking, and standing) depending on on-field positions. For example, forwards spend a larger proportion of time sprinting and cruising compared to mid-fielders or defenders, which is indicative of high intensity interval activity [15]. Mid-fielders spend more time running, reflecting continuous aerobic activity. In contrast, defenders and goalkeepers spend the most time in low intensity activity, with defenders jogging and walking and goalkeepers primarily standing [16]. Sekiguchi et al. (2019) suggested the distance covered is one of the best predictors of hydration status in NCAA Division I soccer players [17]. Thus, the differences in exercise characteristics (intensity and distance) across soccer positions likely requires individualized fluid recommendations and should be accounted for hydration guidelines in a similar way as sport-specific guidelines. 

To our knowledge, no study has examined the position-related differences in overall fluid balance in women’s soccer to assess individualized fluid and electrolyte needs. The purpose of this study was to describe the fluid balance, sweat rate, and electrolyte losses in a single NCAA Division I women’s soccer team. We hypothesized that fluid and electrolyte needs would be dependent on field position with forward and mid-fielders requiring additional fluid intake due to their physical activity pattern. In addition, we examined the effect that environmental conditions had on the relationship between fluid balance and field position. 

## 2. Materials and Methods

### 2.1. Participants

Eighteen players were recruited from a single NCAA Division I women’s soccer team (age: 19 ± 1 yr; weight: 68.5 ± 9.0 kg, and height: 168.4 ± 6.7 cm). Players were grouped according to field position as follows: 3 forwards (FW), 7 mid-fielders (MD), 5 defenders (DF), and 3 goalkeepers (GK). Players were free of any chronic condition and injury and were cleared by their team physician to participate in play. The study was approved by the University Institutional Review Board and all players signed a written informed consent prior to any assessment (Project identification code IRB3627; 3 August 2015).

### 2.2. Experiment Design

The study took place over 7 weeks of practice during a spring off-season training camp (March to mid-April). Players were on-field training every Tuesday and Thursday morning (0730–0900) and Friday afternoon (1530–1700). Due to the accessibility of players and the training schedule, data for this study were only collected on 2 of the 3 practices per week, for a total of 14 evaluated practices. Of the two practices observed, a morning practice (Tuesday and Thursday) and evening practice (Friday) was included. WBGT and relative humidity (RH) varied between 10.2–23.6 °C and 29–86%, respectively. Environmental stress was categorized as a low and moderate risk of hyperthermia using the combination of WBGT and RH [18]. According to Benjamin et al. (2020), a low risk environment for hyperthermia (LOW) was categorized by WBGT < 18 °C and moderate risk (MOD) was categorized as WBGT 18–23 °C with RH ≥ 75% [19]. Practices were monitored on 7 days of LOW and 7 days of MOD risk (Table 1).

Practice consisted of a 10- to 15-min warm-up and 4–5 sets of 15 min scrimmages with a 1- to 2-min break between scrimmages. Study investigators had no influence on the training or sport-specific performance testing. Medical and athletic training staff were present throughout each practice. During the rest period, fluids were provided by the athletic trainers *ad libitum* and consisted of either water and/or carbohydrate-electrolyte solution (CES). Practice assessments involved environmental conditions and physiological monitoring including changes in body mass, fluid intake, urine and sweat assessments, and heart rate (HR). Environmental conditions (WBGT and RH) were measured before and after practice (WBGT8758 Vernon Hills, IL, USA). 

### 2.3. Body Mass

Body mass was assessed using a scale with a precision of 0.1 kg (TANITA TBF-300 Arlington Heights, IL, USA) before and after each practice. Hydration status was represented as %BMC, and fluid balance was calculated as the change in body mass (after toweling dry and urinating), accounting for fluid intake and urine output. In addition, hydration status was categorized by %BMC as euhydrated (EU; ≥0% BMC), mild hypohydration (MI; 0% to −2% BMC), and moderate hypohydration (MO; ≥−2% BMC) to further detect the positional differences [20], despite pre-practice urinalysis (urine specific gravity-USG) suggesting that many players started practice in a fluid deficit (USG > 1.020).

### 2.4. Fluid Intake

Players self-selected beverages to drink *ad libitum* during the practice rest periods. The beverages provided were water (WATER) and/or carbohydrate-electrolyte solution (CES) according to the player’s individual preference during practice. The CES contains 150 mg sodium and 21 g of carbohydrate per serving (360 mL; 80 kcal). Fluid bottles, WATER and CES, were individually labeled with players’ names to ensure an accurate estimate of fluid intake for each player and beverage ingested. Each position and player had equal access to both WATER and CES and the athletic training staff were instructed not to influence beverage selection. Overall fluid intake was determined by pre- and post-practice water bottle weights using food preparation scales (TANITA KD-160BK; Arlington Heights, IL, USA). Players were encouraged to ingest all fluids that entered the mouth so that accurate measures of total fluid intake could be determined. 

### 2.5. Urine Assessments

Players were instructed to completely empty their bladder and provided a urine sample prior to body mass measurements immediately before and after practice. Urine specific gravity (USG) was measured using a spectral refractometer (ATAGO CO., LTD, Bellevue, WA, USA). For the purpose of this manuscript, a USG of ≥1.020 will be considered to be an impaired hydration status as suggested by Casa et al. (2000) [21]. Urine was also analyzed for electrolyte concentrations (sodium (Na+), potassium (K+), and chloride (Cl-)) using ion-selective probes (MEDICA EasyLyte; Bedford, MA, USA).

### 2.6. Sweat Assessments

Sweat was collected on the lower back using the technical absorbent patch technique [22]. The skin surface was cleaned with 70% ethanol and dried before the sweat patch was affixed. Sweat patches were removed 60 min into each practice to avoid patch over-saturation. Regional sweat rate was calculated as the weight difference of the absorbent patch pre- and post-practice divided by the product of the sweat patch area and duration of time the patch was affixed (mg·cm^−2^·min^−1^). Sweat was analyzed for electrolyte concentrations (sodium (Na+), potassium (K+), and chloride (Cl-)) similarly to urine. Regional sweat electrolyte concentrations and sweat rate were corrected to represent whole body losses according to Baker el al. (2019) [23,24].

### 2.7. Heart Rate

Heart rate was monitored continuously using a Bioharness BH3 (Zephyr; Annapolis, MD, USA) worn around the chest during each practice. Eleven of the 18 players wore a Bioharness as designated by the head coach and included FW (*n* = 3), MD (*n* = 5), and DF (*n* = 3). Average heart rate (HRavg) and maximal heart rate (HRmax) were determined for the time during practice. Average exercise intensity was calculated as the HRavg divided by HRmax, multiplied by 100 (%HRmax). HRmax was determined by the maximum HR across 14 days of practice for each player.

### 2.8. Statistical Analysis

**Figure 1:**
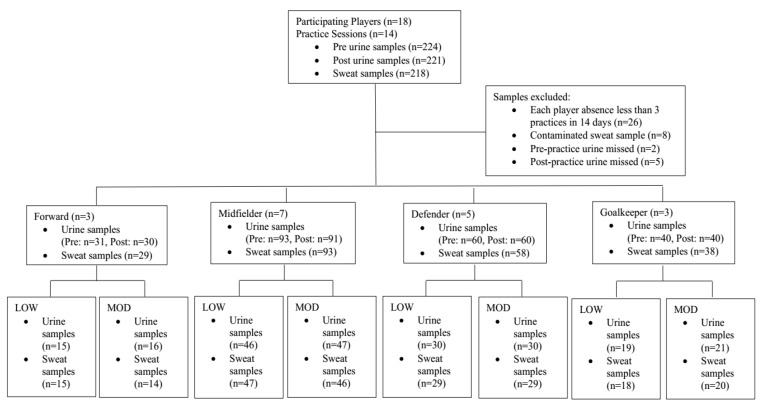
The total sweat and urine samples collected and pooled across 14 practices. Samples were analyzed by position and further categorized by the environmental stress LOW (low risk) and MOD (moderate risk of hyperthermia).

Data were analyzed using JMPro 14 (SAS Inc., Cary, NC, USA). Outcome variables included %BMC, fluid intake, sweat and urine electrolytes, USG, sweat rate, and HR parameters. One- and two-way analysis of variance (ANOVA) were used to analyze outcome variables across field positions (FW, MD, DF, and GK) and environmental conditions (LOW and MOD) with Student-t post-hoc tests to examine group differences. Chi-square (X^2^) was used to analyze BMC categories (EU, MI, and MO) by position. Pearson product moment correlations (r) were used to examine the relationships between outcome variables. The total sample size for sweat and urine assessments is displayed in Figure 1 in 18 players by 14 practices. Twenty-six samples were missed because of absence or because researchers did not have reasonable access to the player. In addition, 8 sweat samples were eliminated due to sample contamination, and 2 pre-training urine samples and 5 post-training urine samples were missed due to additional scheduling conflicts of the players. Data are presented as mean ± standard deviation (SD) and statistical significance was accepted at *p* < 0.05.

## 3. Results

### 3.1. Team Data

Table 2 contains all anthropometric, fluid balance, and sweat and urine analysis outcomes. Mean pre-practice USG was 1.020 ± 0.008 (range 1.003–1.037) with 54% of pre-practice urine samples ≥1.020 (1.022 ± 0.007; range 1.020–1.037) across 14 practices. Pre- and post-exercise USG were not different (*p* = 0.5). Post-exercise urine sodium concentration (111.3 ± 57.4 mmol·L^−1^) was significantly lower compared to pre-exercise urine sodium concentration (144.8 ± 68.6 mmol·L^−1^; *p* < 0.001). Mean %BMC and total fluid intake were −0.5 ± 0.5% and 630 ± 312 g, respectively. Mean sweat rate was 1.03 ± 0.21 mg·cm^−2^·min^−1^ and sweat sodium concentration was 44.5 ± 10.4 mmol·L^−1^. 

### 3.2. Position Data

**Figure 2:**
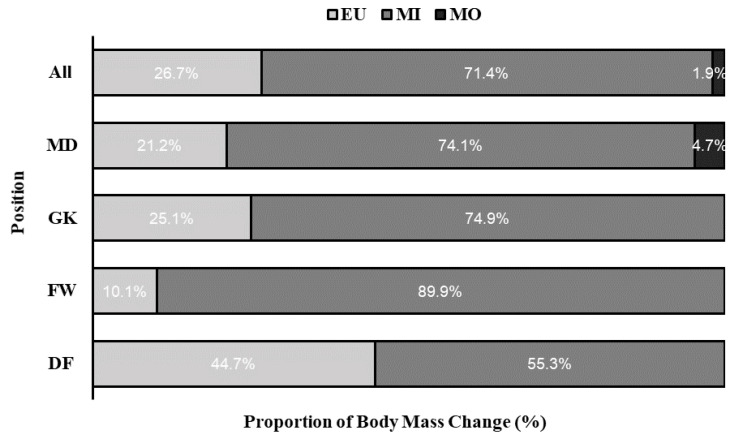
The proportion of body mass change (BMC) overall and within each position across 14 practices. Light grey bars represent no body mass change (EU ≥ 0% BMC); grey bars are BMC <−2% (MI); black bars are BMC ≥−2% (MO). DF—defenders (*n* = 5), FW—forwards (*n* = 3), GK—goalkeepers (*n* = 3), and MD—mid-fielders (*n* = 7).

Categorial hydration status data by position across all practices is displayed in Figure 2. Overall, 26.7% of players were in EU (0.2 ± 0.3%, range: 0.1 to 0.8% BMC), 71.4% of players were MI (−0.8 ± 0.2%, range: −1.9 to −0.2% BMC), and 1.9% MO (−2.2 ± 0.2%, range: −2.5 to −2.0% BMC). Hydration status was statistically different across all positions (X^chi2^ = 21.33, *p* = 0.002).

Markers of fluid balance by field position are also presented in Table 2. %BMC was significantly lower in DF (−0.3 ± 0.6%) compared to FW (−0.6 ± 0.5%), MD (−0.7 ± 0.6%), and GK (−0.5 ± 0.5%, F = 5.38, *p* < 0.001). In addition, DF had the greatest total fluid intake (728 ± 369g; F = 2.97, *p* = 0.03), which was characterized by the greatest CES (525 ± 351g; F = 23.57, *p* < 0.001) and the least WATER (203 ± 264 g; F = 7.25, *p* < 0.001) ingestion compared to other positions. In addition, total fluid intake was significantly associated with %BMC across positions (r = 0.45; *p* < 0.001). GK had significantly lower sweat (Na+) compared to FW, MD, and DF (F = 35.79, *p* < 0.001). FW had significantly greater sweat (Na+) compared to MD and greater sweat (Cl-) compared to MD and DF (*p* < 0.001). Sweat (K+) was greatest in DF compared to FW and MD (*p* = 0.03). However, sweat rate was similar across all practices and all positions (F = 0.94, *p* = 0.4; Table 2). 

### 3.3. Heart Rate Data

An ancillary subset of 11 players wore heart rate monitors during the 14 practices (3 FW, 5 MD, 3 DF). HRavg was similar between positions (FW: 162 ± 17 bpm, MD: 162 ± 14 bpm, and DF: 161 ± 16 bpm; F = 0.07, *p* = 0.9). MD had significantly greater HRmax (216 ± 18 bpm) compared to FW (207 ± 10 bpm) and DF (209 ± 19 bpm; F = 3.23, *p* = 0.04). In addition, FW had a greater average exercise intensity (74.5 ± 8.2% HRmax) compared to MD (67.6 ± 5.8% HRmax) and DF (68.8 ± 7.3% HRmax; F = 8.88, *p* < 0.001). Lastly, exercise intensity (%HRmax) was significantly associated with sweat (Na+) (r = 0.31; *p* = 0.004) across all positions.

### 3.4. Environmental Data

**Figure 3:**
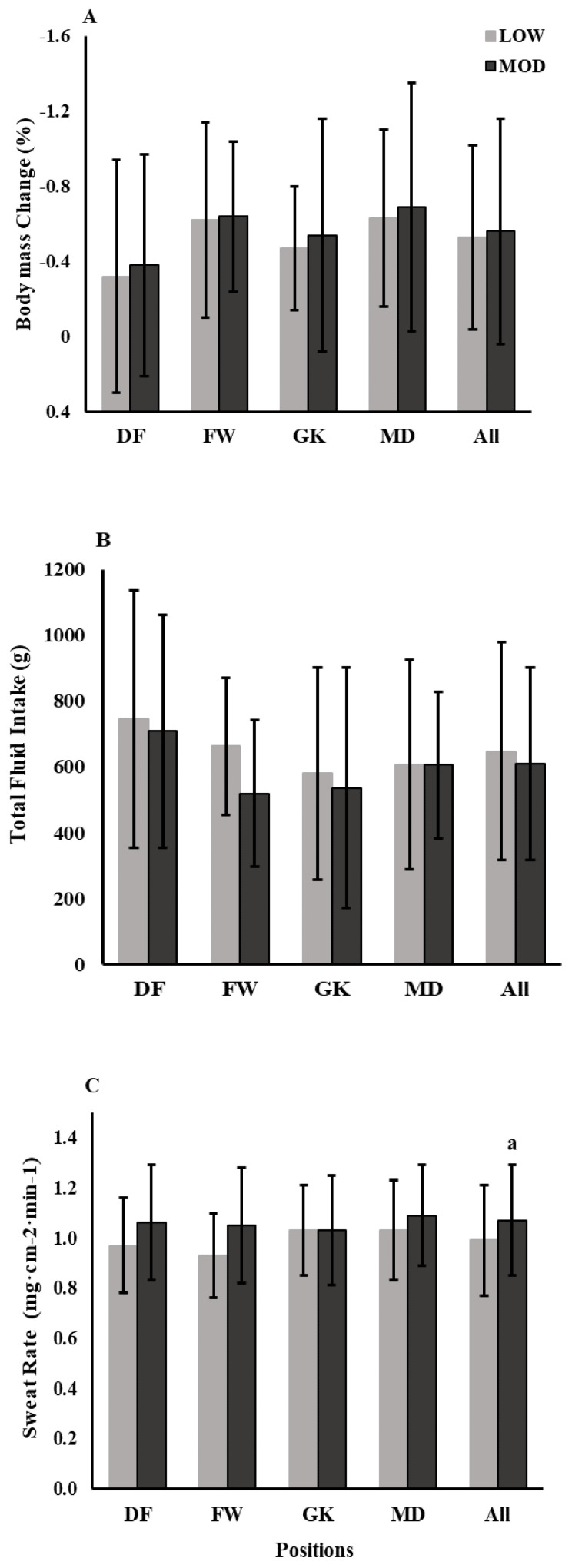
The effects of environments at LOW and MOD risk of hyperthermia on body mass change (**A**), total fluid intake (**B**), and sweat rate (**C**) by different positions. LOW—low risk environment for hyperthermia categorized by WBGT <18 °C (light grey), whereas MOD—moderate risk environment for hyperthermia categorized by WBGT 18–23 °C with RH ≥75% (dark grey). DF—defenders (*n* = 5), FW—forwards (*n* = 3), GK—goalkeepers (*n* = 3), MD—mid-fielders (*n* = 7), and All—across all positions (*n* = 18). ^a^ Significant difference in sweat rate between LOW and MOD environment across all positions (*p* < 0.05).

The averaged WBGT and RH of LOW was 16.2 ± 2.6 °C and 50.5 ± 13.1%, and MOD was 20.5 ± 1.5 °C and 77.5 ± 5.0% (Table 1). No differences existed between the LOW and MOD environmental stress groups for %BMC, total fluid intake, and sweat electrolyte concentrations (all *p* > 0.05). In addition, these outcomes exhibited similar results across all positions in LOW and MOD environments (Figure 3A,B). However, as expected, MOD environment (1.07 ± 0.22 mg·cm^−2^·min^−1^) resulted in a significantly greater sweat rate compared to LOW environment (0.99 ± 0.22 mg·cm^−2^·min^−1^; *p* = 0.02) across all players (Figure 3C).

## 4. Discussion

The present study examined the fluid balance, sweat rate, and sweat and urine electrolyte changes during practice in a women’s NCAA Division I soccer team, considering the differences in physical demands by environmental stress and position. There is a need to re-emphasize pre-practice guidelines for women in sport considering approximately half of the players (54%) began practice in a hypohydrated state. During practice, 71.4% of players had a %BMC < −2%; 26.7% of players had no body mass deficit; and 1.9% of players had %BMC ≥ −2% (all MD position) demonstrating the effectiveness of the players to adequately hydrate during practice. Environments that are considered of a moderate hyperthermia risk significantly elevated sweat rates compared to low risk environments; however, environmental conditions had no influence on fluid intake and hydration status across positions. By position, we observed that DF had a significantly greater total fluid intake and a lower %BMC compared to other positions. FW exhibited greater sweat sodium and chloride concentrations; whereas GK had the lowest sweat electrolyte concentrations compared to other positions. Therefore, positional differences existed in fluid balance and sweat responses, suggesting variable hydration requirements across player positions to maintain both fluid and sodium balance. 

### 4.1. Hydration Status

Players seem to understand the importance of fluid balance during training but may underestimate the importance of pre-practice fluid intake on overall training hydration status. Previous research suggests that USG is a good indicator of pre-practice hydration status, specifically, a USG of >1.020 can be considered chronically hypohydrated [21]. In the present study, 54% of players were hypohydrated prior to the practice (range 25–76% of the players for individual practice). This finding agrees with previous studies [14,25] and suggests that players consumed an insufficient amount of fluid outside of practice. Athletic training staff and coaches should aim to educate players as to the deleterious effects of pre-practice hypohydration to better prepare their players for upcoming practices and competitions [26]. 

The availability of fluids has been suggested to be a key element associated with total fluid intake in team sports [27]. Kilding et al. (2008) found that players were mildly hypohydrated (−0.6% BMC) when consuming 379–450 g of fluids during game-specific training [13]. In addition, Gibson et al. (2012) found that players experienced greater hypohydration (−0.8% BMC) and a total fluid intake less than 250 g [14]. The present study had relatively lower deficits in fluid balance (−0.5% BMC) compared to Kilding et al. (2008) and Gibson et al. (2012), likely due to the greater total fluid intake (~630 g). Unlike the game-specific training in the study by Kilding et al. (2008) and Gibson et al. (2012), which were studies with a minimal opportunity to rehydrate, multiple rest periods in the present study allowed for easier access to fluids. Enhanced fluid accessibility is crucial to maintain overall fluid balance during practice. FIFA and the NCAA are correctly implementing directives, including fluid breaks outside of half time, to encourage proper hydration in soccer matches. 

### 4.2. Positional Differences

Exercise characteristics and running distance vary by position, which are potential factors that affect fluid balance in soccer [17]. In general, elite women’s soccer players cover an average total distance of 8.5 km and an average sprint distance of 14.9 m [28]. The total distance covered also varies by playing position [29,30,31]. For example, Gabbett et al. (2010) found that MD covered ~600 m more than DF and ~1000 m more than FW. This suggests that MD potentially have greater fluid intake requirements compared to other positions [32]. In support of this hypothesis, all of the players with a %BMC ≥ −2% were MD. In addition, MD exhibited the highest maximal heart rates during practice, suggesting high-intensity sprinting during practice. Thus, MD may be at a greater risk of poor fluid balance during practice compared to other positions. In this study, players had equal accessibility to fluids during practice across all positions. Our results demonstrated that MD and FW had less total fluid intake compared to DF. Moreover, a greater proportion of the beverages ingested were CES in DF. This may have resulted in a greater sodium intake in DF and, therefore, better fluid retention and fluid balance compared to other positions [33]. One possible explanation is that MD and FW performed more runs and may not tolerate the ingestion of large volumes of fluids during training, consequently resulting in lower total fluid intake [34]. If this holds true, then pre-practice hydration status and sodium-containing beverage intake are even more important to those soccer players with more running activity like MD and FW to avoid critical hypohydration that may affect performance and place the players at greater risk of heat-related issues. 

Greater sweat sodium and chloride concentrations were observed in FW, whereas the lowest concentrations were found in GK. One possible explanation for this might be that GK had a large proportion of water intake, possibly lowering serum and sweat osmolality, which results in a lower sweat sodium concentration. In addition, sweat sodium concentrations have been shown to be elevated at greater exercise intensities [23]. We examined on-field heart rates to assess exercise intensity in a subset of players. Exercise intensity was higher in FW (75% HRmax) compared to MD (68% HRmax) and DF (67% HRmax). This finding is supported by previous research, suggesting that FW do more sprints and fast runs during practice [29]. On the other hand, GK is considered to be a less-active position compared to other positions, spending a large percentage of time standing. In this study, we found a significant relationship (r = 0.31; *p* = 0.004) between sweat sodium concentration and exercise intensity, suggesting that exercise intensity is a factor associated not only with sweat rate (r = 0.36; *p* = 0.002), but overall sweat electrolyte losses. Given the evidence of the difference in fluid balance outcomes by on-field position, recommendations for fluid intake during training and competition in women’s soccer should be based on on-field exertion characteristics. In this study, the sweat electrolyte concentrations are considered normal in women’s soccer players. Under optimal conditions, an athlete’s sweat rate and electrolyte composition would be known, and with dietary fluid and electrolyte intake, athletic training staff could provide supplemental sodium containing foods or beverages if sodium balance is not achieved. Under normal conditions, athletic trainers and nutritionists should encourage normal dietary intake of fluid and electrolytes; however, supplemental fluid and sodium intake may be encouraged in athletes with high sweat and/or sodium losses, or when athletes have longer practice or practice is conducted under higher thermal stress. Future research could determine intensity thresholds, in combination with environmental conditions, to better estimate fluid intake recommendations across all positions.

### 4.3. Warm and Cool Environments

Estimated sweat rate was similar between positions across all practices. Previous studies have reported that sweat rate cannot be easily explained by fluid intake, pre-exercise hydration status, or variations in body mass within players [35,36]. Our results showed that sweat rate was greater in MOD compared to LOW environment risk categories. We assume that the temperature and water vapor pressure gradients between skin and air are altered during practice in the MOD environmental conditions, initiating cooling mechanisms integrated in the hypothalamus, leading to increased skin blood flow and a greater rate of sweat production for evaporative heat loss [37]. Thus, moderate risk environments seem to elevate sweat rate but similar to other studies, have little influence on fluid intake and hydration status during practice. Future research should investigate positional differences in fluid balance mechanisms across player positions in soccer to verify the altered functional sweating we described in different environmental conditions. These differences could be especially important under higher thermal stress where fluid intake may not offset the higher sweat rates thus possibly producing significant in practice hydration issues. 

A regional sweat patch technique was used to determine regional sweat rate and electrolyte concentrations, which have been shown to represent whole-body sweat electrolyte losses [38]. Mean sweat electrolyte concentrations were similar to previous studies [13,14,39], suggesting the validity of the assessment in this sample. Sweat patches were affixed to the lower back for ease of application and removal by study staff without interfering with practice. The sweat patch was removed within 60 min to prevent over-saturation, which can falsely elevate sweat electrolyte concentrations while underestimating regional sweat rate [40]. Previous research suggests that a single site sweat patch attached to the lower back may overestimate the sweat electrolyte concentrations [41]. While the whole-body wash-down technique is considered the gold-standard assessment of sweat electrolyte losses, this technique is not feasible for on-field assessments in Division I sports that require minimal sweat measurement sites that are easily removable during training to limit practice interruptions. We used a simple method of sweat collection on the lower back as a quick and internally valid method to compare regional sweat rate and electrolyte concentrations within a player on multiple occasions and between a large number of players during on-field team sports. The information gained using this technique has direct real-world applications and should help to improve the wellness and performance of the athletes. 

### 4.4. Strengths and Limitations

Although this study has many strengths including on-field, real-world implications for hydration across diverse environmental conditions, accurate measures of fluid intake by bottle weight changes, repeated measures for each player, and continual assessments of heart rate, and exercise intensity, there are marginal limitations that must be mentioned. First, this study had a small sample size for field-based research, particularly when analyzed by positions. Moreover, although we measured exercise intensity, running distance during training was not recorded, forcing us to make several inferences from other research to describe the players according to position. However, as mentioned above, we did continually assess heart rate responses for a large subset of the players, allowing us to express our results by intensity and time of practice, which should better represent overall effort compared to describing distance alone. In addition, it might not be appropriate to categorize participant’s hydration status after practice as euhydrated, mildly hypohydrated and moderately hypohydrated based on the body mass change because we did not ensure an adequate starting hydration level by forcing fluid intake prior to practice similar to laboratory-based research. This is especially true considering more than half of players started practice in a hypohydrated state according to USG (>1.020). Given this limitation, we applied relative hydration categories to show the magnitude of body mass change to further demonstrate positional differences. A further limitation to this study is the way in which fluid intake was tracked: 2 labeled bottles, 1 WATER and 1 CES, for each player. Normally, each player has a single bottle with their names and beverage of choice. By offering two bottles (CES and WATER) per player at every practice, we may have inadvertently altered drinking behavior [42]. Additionally, similar to previous field-based research conducted by Miller et al. (2020) [24], we used a laboratory-based set of equations to adjust sweat rate and electrolyte concentrations to better represent whole body sweat losses since there are no known practical, real-world, on-field sport equations available. Future research should conduct laboratory and on-field experiments to validate the Baker et al. (2019) [23] equations in settings similar to this study. Lastly, as mentioned in the discussion, we used the technical patch technique to estimate sweat electrolytes and sweat rates. While research has shown that whole-body sweat rate and electrolyte losses are better estimated by whole-body wash down, the real-world on-field nature of this study precludes this methodology.

## 5. Conclusions

In the present study, 54% of players were hypohydrated prior to the practice and the majority of players maintained adequate fluid balance during practice under variable environmental stress. We found the environment to be most likely to influence sweat rate, but not fluid intake and hydration status. By position, DF exhibited a greater total fluid intake, resulting in lower fluid balance deficits. MD appeared to be at the greatest risk of hypohydration with more occurrences of %BMC over −2% and a higher maximal heart rate. FW had the greatest sweat sodium concentrations, whereas, GK had the lowest, suggesting additional sodium should be ingested in more intensive positions. This is the first study to describe the positional differences in fluid balance of collegiate women’s soccer players. These observations in positional fluid balance differences suggest that positional exercise intensity and fluid and electrolyte needs should be considered in future hydration recommendations. In addition, hydration strategies should emphasize pre-practice hydration to ensure that players are prepared for the demands of the sport.

## Figures and Tables

**Table 1:** Data Collection and Environmental Conditions.

	LOW	MOD	All
Practices (*n*)	7	7	14
Pre-urine samples (*n*)	110	114	224
Post-urine samples (*n*)	109	112	221
Sweat samples (*n*)	109	109	218
Ambient temperature (°C)	20.0 ± 2.7	22.3 ± 1.5 ^a^	21.2 ± 2.4
Relative Humidity (%)	50.5 ± 13.1	77.5 ± 5.0 ^a^	64.1 ± 16.7
WBGT (°C)	16.2 ± 2.6	20.5 ± 1.5 ^a^	18.4 ± 3.0

WBGT, wet bulb globe temperature. The WBGT was classified by the low risk (LOW) and moderate risk (MOD) environment for hyperthermia [18,19]. ^a^ Significant difference between LOW and MOD environments (*p* < 0.001).

**Table 2:** Anthropometric, Fluid Balance, and Sweat and Urine Analysis Characteristics of Participants by Field Positions Across Training Practices.

	FW (*n* = 3)	MD (*n* = 7)	DF (*n* = 5)	GK (*n* = 3)	All (*n* = 18)	*p*-Value
Age (years)	20 ± 1	19 ± 1	19 ± 1	19 ± 1	19 ± 1	0.09
Weight (kg)	71.0 ± 6.9 ^b^	63.5 ± 4.9 ^b,d^	63.9 ± 6.0 ^b.d^	82.7 ± 6.1	68.5 ± 9.0	0.001
Height (cm)	169.1 ± 4.9 ^b,c^	165.9 ± 5.1 ^b^	168.0 ± 8.6 ^b,c^	174.4 ± 3.4	168.4 ± 6.7	0.001
Fluid Balance						
Body mass change (%)	−0.6 ± 0.5 ^a^	−0.7 ± 0.6 ^a^	−0.3 ± 0.6	−0.5 ± 0.5 ^a^	−0.5 ± 0.6	<0.001
WATER intake (g)	356 ± 239 ^a^	357 ± 275 ^a,b^	203 ± 264	473 ± 367 ^a^	333 ± 296	<0.001
CES intake (g)	233 ± 248 ^a,b^	242 ± 256 ^a,b^	525 ± 351	80 ± 179 ^a^	288 ± 312	<0.001
%WATER intake	63 ± 31 ^a,b^	60 ± 35 ^a,b^	29 ± 32	79 ± 33 ^a^	55 ± 38	<0.001
%CES intake	37 ± 31 ^a^	40 ± 35 ^a,b^	71 ± 32	21 ± 33 ^a^	45 ± 38	<0.001
Sodium intake (mg)	97 ± 103 ^a,b^	100 ± 106 ^a,b^	217 ± 145	33 ± 74 ^a^	119 ± 129	<0.001
Total fluid intake (g)	589 ± 225 ^a^	607 ± 273 ^a^	728 ± 369	559 ± 340 ^a^	630 ± 312	0.03
Urine Analysis						
Pre USG	1.023 ± 0.007^c^	1.017 ± 0.009	1.021 ± 0.007 ^c^	1.020 ± 0.007	1.020 ± 0.008	0.002
Pre (Na^+^) (mmol·L^−1^)	178.5 ± 64.8	133.1 ± 72.4 ^d^	141.5 ± 59.0 ^d^	151.4 ± 69.3	144.8 ± 68.6	0.02
Pre (K^+^) (mmol·L^−1^)	42.8 ± 17.0	43.0 ± 31.0	51.4 ± 27.7	56.8 ± 39.9	47.7 ± 30.7	0.07
Pre (Cl^−^) (mmol·L^−1^)	158.4 ± 57.8	128.0 ± 71.9	132.2 ± 54.8	141.8 ± 75.9	135.7 ± 66.9	0.16
Post USG	1.021 ± 0.008 ^c^	1.017 ± 0.008	1.022 ± 0.006 ^c^	1.021 ± 0.007 ^c^	1.020 ± 0.008	0.001
Post (Na^+^) (mmol·L^−1^)	143.2 ± 54.7	96.6 ± 55.6 ^a,d^	123.1 ± 58.5	104.9 ± 50.0 ^d^	111.3 ± 57.4	0.001
Post (K^+^) (mmol·L^−1^)	57.6 ± 24.9	50.0 ± 31.8	58.1 ± 22.9	60.7 ± 30.4	55.1 ± 28.6	0.16
Post (Cl^−^) (mmol·L^−1^)	140.6 ± 57.3 ^c^	95.1 ± 58.3	120.9 ± 55.2 ^c^	121.4 ± 65.6 ^c^	112.7 ± 60.5	0.001
Sweat Analysis						
Sweat (Na^+^) (mmol·L^−1^)	51.4 ± 9.8 ^b^	46.0 ± 9.4 ^b,d^	48.0 ± 6.4 ^b^	30.9 ± 3.9 ^d^	44.5 ± 10.4	<0.001
Sweat (K^+^) (mmol·L^−1^)	4.9 ± 1.4 ^a^	5.3 ± 0.9 ^a^	5.8 ± 1.3	5.3 ± 1.0	5.4 ± 1.1	0.03
Sweat (Cl^−^) (mmol·L^−1^)	47.6 ± 9.4 ^b^	42.1 ± 9.0 ^b,d^	43.1 ± 6.4 ^b,d^	29.9 ± 3.3 ^d^	40.5 ± 9.9	0.001
Sweat rate (mg·cm^−2^·min^−1^)	0.99 ± 0.21	1.06 ± 0.21	1.02 ± 0.21	1.03 ± 0.20	1.03 ± 0.21	0.42

FW, forwards; MD, midfielders; DF, defenders; GK, goalkeepers; CES, carbohydrate-electrolyte solution; USG, urine specific gravity. ^a^ Significantly different compared to DF (*p* < 0.05). ^b^ Significantly different compared to GK (*p* < 0.05). ^c^ Significantly different compared to MD (*p* < 0.05). ^d^ Significantly different compared to FW (*p* < 0.05).
